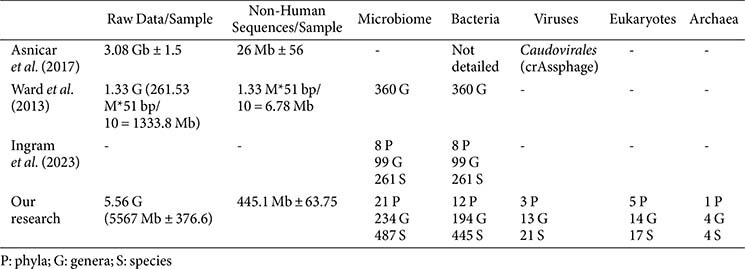# Microbiome Profiling of Pretreated Human Breast Milk Using Shotgun Metagenomic Sequencing

**DOI:** 10.4014/jmb.2506.06012

**Published:** 2025-10-28

**Authors:** Qiao Zhang, Yi Zhang, Jianjiang Zhu, Yajun Gao, Wen Zeng, Hong Qi

**Affiliations:** 1 Department of Prenatal Diagnosis, Haidian District Maternal and Child Health Care Hospital, Beijing 100080, P.R. China; 2 Department of Prevention and Treatment of Breast Diseases, Haidian District Maternal and Child Health Care Hospital, Beijing 100080, P.R. China

**Keywords:** Human breast milk, metagenomic sequencing, nucleic acid extraction, wall breaking parameter, High-Throughput Nucleotide Sequencing

## Abstract

This study explored the metagenomic sequencing methodology for analyzing the breast milk microbiome and elucidated its composition. Twenty-two breast milk samples were collected from 11 healthy lactating women. By optimizing microbial cell wall disruption parameters and developing a nucleic acid extraction method, microbial DNA/RNA libraries were constructed and subjected to metagenomic next-generation sequencing (mNGS), microbial standards spiked into breast milk at serial dilutions served to validate the method’s reliability. The sequencing data underwent rigorous quality control and classification using the Kraken2 software and a self-generated database. The breast milk microbiome was found to comprise 21 phyla, 234 genera, and 487 species, with *Firmicutes* and *Proteobacteria* being the dominant phyla. At the genus level, *Staphylococcus* and *Streptococcus* were the most abundant, while at the species level, *Staphylococcus aureus*, *Streptococcus bradystis*, and *Staphylococcus epidermidis* were the most prevalent. The microbial profiles of the left and right breast milk samples were consistent at the phylum, genus, and species levels. Besides common bacteria, diverse viral, eukaryotic, and archaeal sequences were also detected. Functional profiling revealed that the "lactose and galactose degradation I" pathway accumulated the highest read count, whereas the L-valine biosynthesis pathway was detected most frequently. This study provides a comprehensive understanding of the healthy breast milk microbiome, highlighting the presence of specific flora colonization and the distinct yet correlated microbial environments in bilateral breast milk, laying the groundwork for future research into the interactions between breast milk microbiota and maternal and infant health outcomes.

## Introduction

Breast milk microbiota and its metabolites contribute positively to the development of gastrointestinal microbiota, regulating host gene expression and supporting immune development in infants. Breastfeeding is a critical determinant of the composition and functionality of the gastrointestinal microbiome of an infant [1]. Understanding the human milk microbiome and implementing targeted beneficial interventions for developing infants to optimize the microbiome in breast milk, donor milk, or formula can improve maternal and infant health throughout their lifespan. Additionally, investigating breast milk microbiota contributes to advancements in diagnosing infectious diseases with greater accuracy and efficiency [2, 3].

Microbiome research uses several techniques, including bacterial culture, amplicon sequencing, and mNGS [4, 5]. Culture-based methods typically rely on specific nutrient media and environmental conditions to cultivate microorganisms, allowing direct observation and analysis of their morphological, physiological, and biochemical characteristics. However, this approach is limited because only a portion of culturable microorganisms can be detected and does not capture the full diversity of the microbial community [6-9].

Amplicon sequencing uses second/third-generation sequencing platforms to analyze targeted PCR products, such as 16S rRNA (ribosomal RNA), 18S rRNA, ITS (internal transcribed spacers), or functional genes. This approach addresses the drawbacks of traditional culturing by identifying non-culturable microorganisms and providing insights into the microbial community structure, evolutionary relationships, and their environmental correlations. It can directly detect the presence of microorganisms in the environment and is suitable for investigating rare or difficult-to-culture microorganisms. In recent years, 16S rRNA sequencing technology has been widely utilized to study the breast milk microbiome [4, 10]. However, this method has certain limitations. Different hypervariable target regions of 16S rRNA can yield distinct phylum profiles from the same sample, and it is difficult to detect novel or highly variable microorganisms, such as viruses and fungi. Additionally, the limited length of amplicons restricts the taxonomic resolution at the genus and species levels [11, 12]. Compared with the 16S rRNA sequencing method, the 16S-ITS-23S rRNA sequencing method can amplify a 2,500 bp fragment in a single-step PCR, offering higher resolution for studying milk bacteria. However, it detected fewer bacteria than the 16S rRNA sequencing method [13]. In contrast, mNGS offers an unbiased and comprehensive method to profile all microbial DNA/RNA in a sample, without the need for targeted primers. It enables simultaneous detection of bacteria, viruses, fungi, and archaea, and can potentially identify emerging pathogens, resistance genes, and strain-level variations [8, 14]. The clinical value of mNGS has been demonstrated in detecting pathogens in complex samples like cerebrospinal fluid and plasma [7], and its application in low-biomass environments like breast milk may significantly improve understanding of maternal–infant microbial transfer and diagnostic microbiology [9]. However, to date, only a few studies have applied mNGS to breast milk, and most lack optimized pretreatment methods to enhance microbial recovery and sequencing efficiency [15, 16].

One of the key challenges is that considerable fat, protein, and protease content in breast milk interfere with microbial detection, reducing the efficiency of microbial extraction and analysis. Recent studies have shown that the breast milk microbiome differs significantly between mothers of term and preterm infants, with preterm-associated milk often showing reduced microbial diversity and altered abundance of key commensals such as Staphylococcus, Streptococcus, and Bifidobacterium [17, 18]. These microbial differences may influence neonatal outcomes, especially in vulnerable preterm populations. Moreover, given the low microbial biomass and high lipid/protein content of human milk, recent efforts have been made to improve nucleic acid extraction protocols, often involving bead-beating, enzymatic lysis, or chemical disruption steps to improve recovery from both gram-positive bacteria and fungi [12, 19]. Some researchers have optimized and applied methods for detecting phages in breast milk and infant feces, reducing the required breast milk sample volume to 0.1 mL [20].

Nevertheless, no gold-standard protocol yet exists; most current approaches recover DNA quantities inadequate for high-resolution profiling and routinely miss non-bacterial constituents. These gaps motivated us to refine a milk-specific pretreatment/extraction pipeline that reliably captures the full spectrum of microbial DNA.

In this study, by integrating this optimized pipeline with deep shotgun metagenomic sequencing, we systematically resolved the microbial composition and functional potential of healthy human breast milk across phylum, genus, and species levels, delivering the high-resolution, domain-wide portrait of the milk microbiome.

## Materials and Methods

### Research Objects and Sample Collection

**Research object.** Twenty-two milk samples were collected from 11 breastfeeding women at the Haidian District Maternal and Child Health Hospital in 2023 at 42 days postpartum. Basic information was provided in the supplemental material (Table S1). All participants were healthy, with no signs of infection, such as fever, incision infections, mastitis, or endometritis, before and after delivery. The participants had no pregnancy complications, such as pregnancy-induced hypertension syndrome, severe anemia, or intrauterine growth restriction. None of the participants had consumed antibiotics or probiotics within 1 month of delivery. The study was conducted in accordance with the Declaration of Helsinki and approved by the Ethics Committee of Beijing Haidian District Maternal and Child Health Hospital (Approval Code No. 2024-10, Approval date February 8, 2024). All participants provided signed informed consent, which was approved by the local ethics committee.

**Milk collection.** Following thorough iodophor disinfection of the nipple and surrounding areola, the initial 1–2 ml of foremilk was discarded to reduce the potential contribution of surface-associated or duct-resident microorganisms. A total of 5 ml of midstream milk from each side was then collected into sterile tubes. All procedures were conducted under aseptic conditions by trained nurses. Samples were immediately stored at -20°C for downstream analysis.

### Methods

**Establishment of sample pretreatment method.** *Candida albicans* (Hebei Beina Biotechnology Co., Ltd., China), *Streptococcus agalactiae* (Hebei Beina Biotechnology Co., Ltd.), and *Klebsiella pneumoniae* (Hebei Beina Biological Technology Co., Ltd.) were selected as representative fungi, gram-positive cocci, and gram-negative bacilli, respectively. These cultures were mixed, and multiple glass beads, zirconium beads, and quartz sand ternary wall-breaking tubes were used and compared with a single pickling glass wall-breaking tube.

The parameters for the wall-breaking instrument, including oscillation speed (S), running time (T), interval time (D), and cycle number (C), were systematically adjusted. Nucleic acids were extracted and amplified using real-time quantitative PCR. Optimal wall-breaking parameters corresponding to the minimum and lowest cycle threshold (CT) value dispersions of the three microorganisms were selected by considering the mean and standard deviation (SD) of the CT value. In the wall-breaking system, Bead Ruptor 12 (OMNI, USA) was used for pretreatment. Gradients of the wall-breaking parameters are listed in Table S2 in the supplemental material file.

The microbial community standard (Zymo Research, USA) was spiked into breast milk and diluted 10-fold to obtain three concentration gradient samples of low(L), medium(M) and high(H) concentration, with saline as the negative control, and then metagenomic sequencing was performed. The reliability of the detection method was evaluated according to the quantitative results of each strain.

**Nucleic acid extraction.** A volume of 800 μl of each milk sample was transferred to a 1.5 ml nuclease-free tube and centrifuged at 13,800 ×*g* for 5 min to separate the milk fat layer. About 400 μl of the infranatant is aspirated into a fresh 2.0 ml nuclease-free tube and set aside, after which the remaining about 200 μl of pellet was transferred into a grinding tube with strict avoidance of the cream layer. All supernatant was retained, and only the pellet was subjected to bead-beating following the optimized parameters earlier described. After lysis, the retained supernatant was returned to the grinding tube and the mixture is thoroughly resuspended.

After the samples were pretreated, nucleic acids were extracted using the VAMNE Magnetic Pathogen DNA/RNA Kit (Vazyme Biotech Co., Ltd., China) and quantified using Qubit 3.0 (Invitrogen, USA).

**Paired-end sequencing library construction and high-throughput sequencing.** Microbial DNA/RNA libraries were constructed using 30 ng of nucleic acids with the Rapid Max DNA Lib Prep Kit for Illumina (ABclonal, China). After library construction, the concentration was measured using a Qubit 3.0 fluorescence quantifier, confirming a concentration exceeding 0.1 ng/μl. Paired-end sequencing was performed using the Illumina NovaSeq6000 platform, generating 2 × 150 bp short reads per sample. Raw reads were filtered for quality and host contamination prior to taxonomic classification, generating a data volume of 20 million reads per sample.

### Data Analysis

Bowtie2 was used to compare the original filtered sequence with the human reference genome (hg19). The human source sequences were excluded, and the k-mer LCA method was used to compare species with the self-owned data generated using the Kraken2 software. A proprietary database was constructed using Kraken2 for species comparison. This database included all bacterial, archaeal, fungal, protozoan, and viral species in the NCBI RefSeq database (Release 210, January 7, 2022), comprising approximately 19,400 species, including common pathogens. Data analysis was performed using QIIME Version 2:2020.2.0 software [21]. Species abundance was calculated by RPM (the number of organism reads per million sequence reads, Taxon Reads × 10^6^ / Total Reads) represented by the formula: The total number of reads should be employed under appropriate circumstances to standardize the total sequence number of samples, which is conducive to inter-sample comparison and positive determination. To evaluate whether sequencing depth was sufficient to capture microbial diversity, rarefaction curves were generated for each sample using species-level read count data. To assess inter-individual microbial variation, genus-level read count data were log-transformed and used to generate a hierarchical clustering heatmap based on Bray-Curtis dissimilarity using R (v4.2.0) and the pheatmap package. Samples were grouped based on relative taxonomic abundance across all domains. Species with low abundance across all samples were filtered out for clarity.

To perform functional annotation of these identified molecules of metageomics origin, filtered reads of all individual samples were then given to methPhlan and HUMAnN to realign to known UniRef protein sequences. Pathways involving those proteins with high abundance of mapped reads were aggregated across organisms and annotated using MetaCyc database. The per-library pathway abundance was then normalized by the total read number of the sample and multiplied by 1 million.

### Statistical Analysis

Mean and SD calculation of CT values for wall disruption in pretreated milk samples from healthy lactating women and total sequence data were performed using SPSS v22.1 (IBM, USA).

## Results

### Optimization of Human Milk Metagenomic Sequencing Methodology

**Sample pretreatment.** The mean and SD of two CT values were analyzed in relation to the wall-breaking method used for pre-treated samples. Detailed CT data are provided in the supplemental material (Table S3). When compared with the single-pickling glass tube, the CT and mean values for the ternary wall-breaking tube were lower. Based on the wall-breaking parameters listed in Table S2, the lysis efficiency of the bacterial mixture was evaluated by systematically varying four variables—oscillation speed (S), running time (T), interval time (D), and cycle number (C)—to generate scheme 1–8. Scheme 5 employed the ternary wall-breaking tube (glass, zirconium, quartz sand) with an oscillation speed of 4.0 m/s, 3 cycles of 30 s with 10 s intervals, determined to yield the lowest CT values for all target organisms (Table 1). Based on these results, subsequent experiments were conducted using the wall-breaking method of the ternary wall-breaking tube, as described in Scheme 5.

**Microbial detection of reference standard into breast milk at different dilution gradients.** To further verify the feasibility of the method, microbial standards were spiked into breast milk at serial dilutions and subjected to metagenomic sequencing. The Table 2 showed that the detected abundance was positively correlated with the theoretical concentration. Only *Staphylococcus aureus* was disturbed due to the high background of breast milk.

### Metagenome Sequencing of Healthy Maternal Breast Milk

**Species distribution at phylum, genus, and species levels in milk of healthy mothers.** The total sequence data size of the samples was 5,567 ± 376.6 Mb, while the non-human sequences was 445.1 ± 63.75 Mb. Details of the statistics for the effective sequence data and rarefaction curves of each sample are included in the supplemental material (Table S4, Fig. S1).

**Fig. 1:**
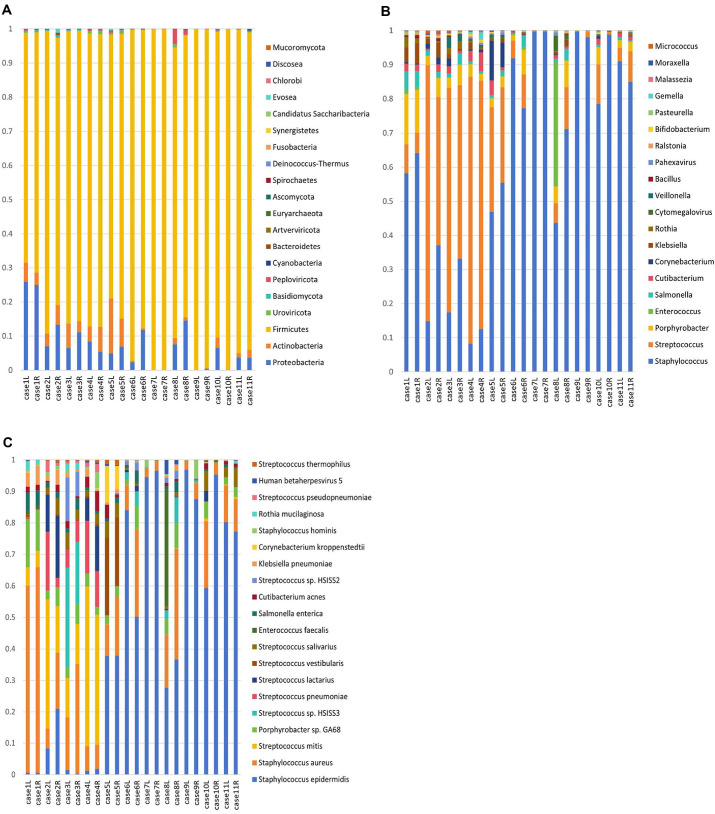
Microbial species distribution in the breast milk of healthy lactating women, depicted at the phylum, genus, and species levels. (**A**) Top 20 abundant phyla in breast milk. (**B**) Top 20 abundant genera in breast milk. (**C**) Top 20 abundant species in breast milk.

Microorganisms identified in the breast milk of healthy mothers were classified into 21 phyla, 234 genera, and 487 species. Analysis of the top 20 abundant phyla, genera, and species revealed that *Firmicutes* predominated at the phylum level, followed by *Proteobacteria*, which collectively accounted for more than 90% of the bacterial species (Fig. 1).

Besides common bacterial species, this study identified *Euryarchaeota* and detected fungi, including *Candidatus*, *Saccharibacteria*, *Mucoromycota*, and *Planctomycetes*. Additionally, viruses such as *Peploviricota*, *Uroviricota*, and *Artverviricota* were also present.

At the genus level, *Staphylococcus* and *Streptococcus* constituted most of the microbiota. *Cytomegalovirus* and *Pahexavirus* virus were detected within the top 20 genera. Furthermore, *Malassezia* was prevalent in milk. *Bifidobacterium* was extensively present, while small quantities of *Lactobacillus* were observed in some individuals.

*Staphylococcus epidermidis*, *S. aureus*, and *Strepococcus mitis* accounted for most of the microorganisms at the species level. The phylum and genus levels were largely consistent on both sides. However, significant variability was observed at the species level among different individuals.

**Fig. 2:**
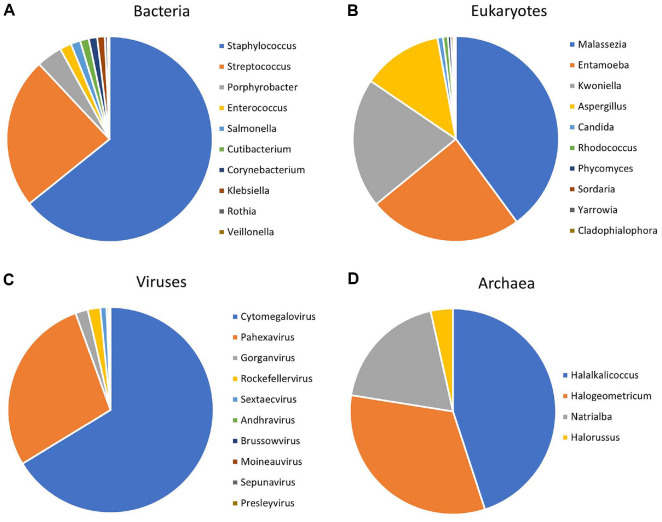
Relative abundances of dominant genera in breast milk across different microbial domains. (**A**) Top 10 bacterial genera. (**B**) Top 10 eukaryotic genera. (**C**) Top 10 viral genera. (**D**) Top 10 archaeal genera.

**Compositions of dominant genera in breast milk across different microbial groups.** To enhance data visualization and better illustrate the diversity across different microbial domains, we constructed separate genus-level relative abundance pie charts for bacteria (Fig. 2A), eukaryotes (Fig. 2B), viruses (Fig. 2C), and archaea (Fig. 2D). These charts highlight the most abundant genera within each microbial domain across all samples.

**Alpha diversity index analysis.** Alpha diversity serves as a comprehensive indicator of community richness and evenness. The Chao1 index, which measures community richness, and the Shannon Index, which assesses both community richness and uniformity, were used to evaluate the diversity of microbial communities in milk (Table 3). The Chao1 index ranged from 59 to 216.6, whereas the Shannon index varied from 0.23 to 3.88. Alpha diversity showed fluctuations across samples. The number and abundance of species on the left and right sides of Case 3 milk exhibited significant dissimilarity. In contrast, the abundance and evenness of microbial communities were consistent between the left and right sides of the other samples.

**Fig. 3:**
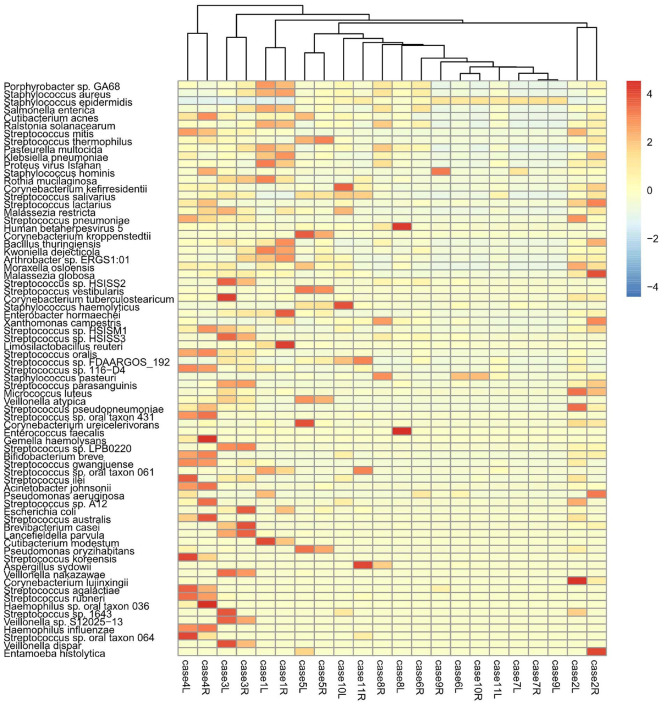
Heatmap of microbial species abundance across all breast milk samples. Hierarchical clustering of the 22 milk samples (11 participants, left and right sides) based on the log-transformed relative abundance of the top 70 microbial species. Clusters illustrate both conserved and participant-specific microbial signatures.

**Inter-individual variability in microbial composition.** To evaluate the variation in microbial communities across the 11 participants, a heatmap was constructed using log-transformed relative abundances of the top 70 species across all breast milk samples (Fig. 3). The clustering analysis revealed both shared and individual-specific microbial features. For example, *S. aureus*, *S. mitis*, and *S. epidermidis* were consistently abundant in most samples, whereas *Malassezia restricta*, *Kwoniella dejecticola*, and *Entamoeba histolytica* appeared only in specific individuals. Some participants (*e.g.*, case 3, case 6) exhibited unique microbial profiles that were distinguishable from other samples, particularly in the viral and eukaryotic domains. These results highlight the presence of a core breast milk microbiome shared across individuals alongside subject-specific variations that may be influenced by maternal physiology, environment, or diet.

**Comparative analysis of metagenomic sequencing in healthy breast milk.** Compared with existing studies on the metagenomic analysis of microorganisms in healthy human breast milk using the Illumina sequencing platform, the average yield of our samples was 5,567 ± 376.6 Mb, of which 445.1 ± 63.75 Mb originated from non-human reads. This yield is considerably higher than the values reported in the other metagenomic studies. Additionally, various viruses, eukaryotes, and archaea were identified in breast milk. The sample pretreatment method significantly enhanced the sensitivity of Illumina metagenomic sequencing, enabling a more detailed classification of microorganisms at the phylum, genus, and species levels (Table 4).

**Fig. 4:**
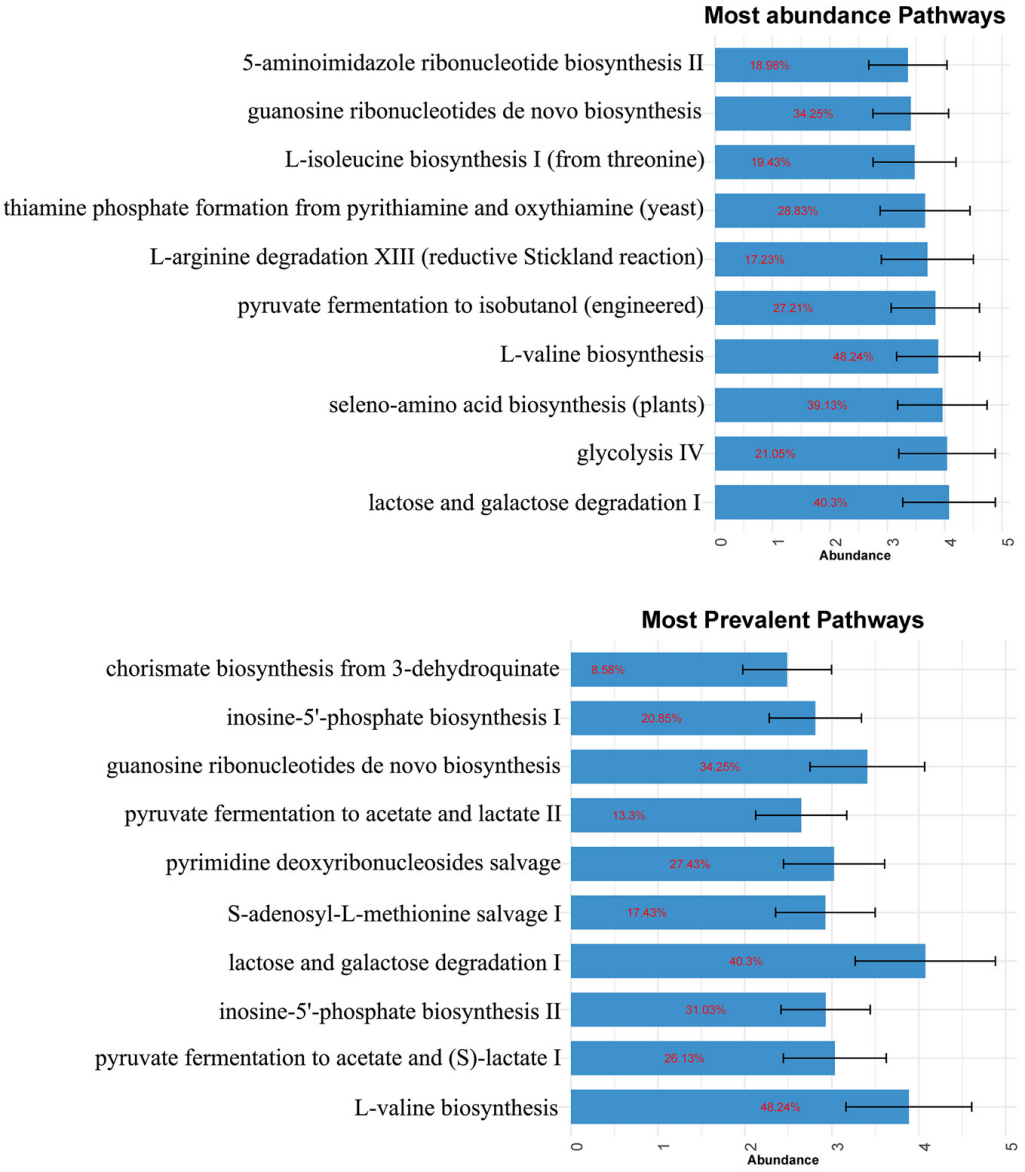
Abundance of top 10 enriched functional pathways in terms of abundance (left) and prevalence (right). The pathway information was taken from MetaCyc database. Error bars represent the population level standard errors of the mean abundance of each pathway. The red numeric value signifies the pathway coverage.

**Metabolic functional potentials of the microbiome in healthy breast milk.** In Fig. 4, potentially enriched pathway of those top-ranking microbiomes was investigated. The pathway which accumulates the most readout is ‘lactose and galactose degradation I’, which is responsible for the initial hydrolytic cleavage of lactose to glucose and galactose by β-galactosidase. The most frequently identified pathway is ‘L−valine biosynthesis’, which is responsible for the biosynthesis of three essential amino acids of vertebrates, isoleucine, valine and leucine. Other frequently found and also abundant pathways include various forms of pyruvate fermentation, to acetate and lactate, which is an essential process for energy metabolism. The molecules contributing to these pathways are mostly originated from the genus of *Staphylococcus* and *Streptococcus*.

## Discussion

In this study, gradient centrifugation was used to effectively remove a substantial quantity of milk fat from breast milk. Subsequently, a ternary cell wall disruption tube, composed of glass beads, zirconium beads, and quartz sand, was used to enhance mechanical shear forces on complex microbial cell walls. This approach achieved a more comprehensive cell wall disruption effect than a single pickling glass cell wall disruption tube. Following optimization of the cell-wall disruption parameters, the extraction efficiency for fungi and certain bacteria with challenging cell walls to disrupt was markedly improved, facilitating the maximum extraction of complete microbial nucleic acids. Subsequently, the quantitative microbiome standards were added to breast milk for gradient dilution, and metagenomic sequencing was performed to verify the reliability of the detection method. The spiking experiment showed that except for *S. aureus*, the changes of other microorganisms were consistent with the expected trend, presumably due to the excessive abundance of *S. aureus* in the breast milk matrix.

Building on these effective pretreatment methods, metagenomic sequencing technology was applied to detect and analyze milk microorganisms, including bacteria, archaea, eukaryotes, and viruses. Compared to the study by Ward *et al*. [22], which analyzed only prokaryotes in breast milk at the phylum and genus levels, this study extended microbial detection to the species level. While Ingram *et al*. [23] analyzed microbes in breast milk across the phylum, genus, and species levels, they exclusively detected bacteria and identified a limited number of microorganisms. In this study, we have achieved a significant enhancement in sequencing efficiency, with the average non-human sequence data (445.1 Mb) vastly surpassing that of Asnicar *et al*., who reported 26 Mb [15]. This study identified archaea, eukaryotes, and viruses in addition to common bacterial species in healthy breast milk, consistent with recent reports on milk microbial diversity [3].

At the phylum level, the types of bacteria identified were largely consistent with those reported in the literature; however, slight variations in composition were observed. *Proteobacteria* recorded the highest abundance in previous studies, while *Firmicutes* and *Proteobacteria* exhibited the highest abundance in the horizontal distribution, collectively accounting for more than 90% of the total bacterial species in healthy breast milk with *Firmicutes* recording the highest abundance. These differences could be attributed to regional variations, sampling times, and variations in data analysis methods [11, 22].

In this study, *Staphylococcus* and *Streptococcus* were confirmed as the dominant genera at the genus level, consistent with findings from other non-culture-based methods, such as rRNA PCR, gas chromatography/mass spectrometry, and 16S rRNA sequencing [24, 25]. In contrast, the proportion of *Pseudomonas* was relatively low in this study, with an average of only 0.09%. However, previous reports indicate that, besides *Staphylococcus* and *Streptococcus*, *Pseudomonas* are present in considerable numbers [11, 22]. The variability in findings related to *Pseudomonas* remains controversial, with a few studies suggesting that *Pseudomonas* can be isolated from any sample, while others speculate that its high abundance may result from DNA extraction kits or other laboratory reagents [26, 27]. We performed an in-depth analysis of the top-ranking microbial enrichment pathways and found that most molecules within these pathways originated from *Staphylococcus* and *Streptococcus*, further suggesting their potential probiotic roles during the early establishment of the neonatal gut microbiota.

Species-level analysis revealed remarkable individual variation, likely related to maternal geographical location, diet, and lifestyle. Despite this variation, *S. epidermidis* and *S. aureus* were consistently identified as the predominant species in healthy breast milk. These findings align with previous species-level studies using both culture-based and non-culture-based methods [3, 4, 12]. Our clustering heatmap further supports the presence of both core and variable microbiota across individuals. While dominant genera like *Staphylococcus* and *Streptococcus* were detected in nearly all participants, substantial differences in fungal, archaeal, and viral taxa were noted. This inter-individual variability has also been observed in previous studies [17] and may be shaped by maternal factors, including delivery mode, lactation stage, and immune status. Our findings reinforce the complexity and individuality of breast milk microbiomes, especially when analyzed at species-level resolution.

Besides common bacteria, this study identified various other microorganisms in breast milk. Viruses, including *Uroviricota*, *Peploviricota*, and *Artverviricota*, were detected. Among fungi, *Basidiomycota* and *Ascomycota*, which have been reported previously, were detected along with *Discosea* and *Mucoromycota*. Additionally, an archaeon of the *Euryarchaeota* phylum was detected. Although individual viruses, fungi, and archaea were detected using other methods, our findings expand upon the limited and simplistic microbial classifications in earlier reports [2, 3].

Although many of the microorganisms identified belong to the same phylum as those of prior studies, considerable differences were observed at the genus and species levels [2, 3]. Togo *et al*. [16] reported the presence of methanogenic archaea in breast milk. The distinction is that the Halobacteriaceae archaea identified in our study are consistent with those detected using 454 pyrosequencing; however, they represented distinct species categories [11]. Additionally, our study is the first to report the presence of protozoan *Evosea* in breast milk.

Through microbial diversity analysis, the Chao1 index range in healthy maternal milk was determined to be 59–216.6, while the Shannon index ranged from 0.23 to 3.88. Differences in alpha diversity among individuals may be associated with variations in age, diet, geographic region, and work schedules of pregnant women. While the diversity of microflora in the left and right milk of participants was generally similar, the number, richness, and evenness of the microflora were not entirely identical. In Case 3, the microflora diversity on the left side differed markedly from that on the right. This observation suggests that although the microbiota of the left and right sides is related, they may exhibit independent characteristics.

Although precautions such as iodophor disinfection and foremilk removal were applied to minimize contamination from nipple or skin flora, we acknowledge that complete exclusion of external microorganisms cannot be guaranteed, which represents a limitation of our study. Several studies have shown that skin-associated genera such as *Staphylococcus*, *Corynebacterium*, and *Propionibacterium* are frequently detected in both milk and on the nipple surface [11, 28]. However, in our dataset, we also observed high abundances of milk-intrinsic genera such as *Streptococcus*, *Bifidobacterium*, and *Malassezia*, which are less commonly associated with skin microbiota. Furthermore, compared with skin microbiome studies, our data revealed a higher diversity of anaerobic and gut-origin taxa (*e.g.*, *Bifidobacterium*, *Lactobacillus*), as well as the presence of eukaryotic and viral sequences that are rarely reported in nipple skin studies [6]. These findings suggest that while some degree of surface contamination cannot be ruled out, the majority of microbial DNA identified likely originates from the milk itself, and our protocol aligns with accepted practices in the field.

The sample size of this study was relatively small, comprising only 11 participants. This limitation restricts the generalizability of the findings to broader populations. The small sample size may also reduce the statistical power to detect subtle differences or associations within the data. Future research should aim to recruit a larger and more diverse cohort to validate these preliminary results and to provide more robust insights into the breast milk microbiome and its potential impact on maternal and infant health.

Microbial loss may have occurred during centrifugation and fat layer removal, particularly for viruses and microorganisms that partition with lipids. Recent studies have confirmed the presence of cells and DNA in the milk fat fraction [29]. However, the presence of milk fat significantly hinders the extraction of nucleic acids from breast milk. While our approach improved sequencing efficiency, it may under-represent certain milk-associated taxa.

Besides sample-related limitations, the mNGS methodology itself presents certain constraints. Due to its extremely high sensitivity and unbiased detection capability, mNGS can identify a wide range of microbial taxa, including environmental or commensal microbes not necessarily associated with disease. This poses a challenge for clinical interpretation, as distinguishing true pathogens from background or contaminating organisms requires careful contextual analysis, especially in low-biomass samples such as breast milk. Moreover, while our sequencing depth enabled broad taxonomic profiling, it may not have been sufficient to confidently detect low-abundance functional genes, such as antimicrobial resistance (AMR) genes or virulence factors. These elements typically require targeted enrichment or ultra-deep sequencing to be accurately quantified. Previous studies using targeted metagenomics, such as 16S rRNA or ITS sequencing, typically report milk microbiota at the phylum and genus levels, with *Firmicutes*, *Proteobacteria*, and Actinobacteria as dominant phyla [4, 17]. However, these methods are inherently limited by PCR biases and primer specificity, which can lead to the underrepresentation of fungi, archaea, or viruses. In contrast, our shotgun-based approach allows the detection of all microbial domains. Nonetheless, the limited read length (150 bp) restricts accurate classification at the species level, which aligns with findings from previous studies cautioning against overinterpretation of species-level calls using short-read data [21]. Importantly, our data corroborate previous reports in terms of dominant bacterial taxa but also expand on viral and fungal diversity not captured by amplicon-based approaches.

In summary, the pretreatment method established in this study markedly enhanced the detection capability of the metagenomic sequencing method. This approach enables the direct and effective extraction of DNA/RNA from all microbial genomes in breast milk for sequencing. Consequently, it facilitates a more comprehensive analysis of the diversity and functional characteristics of the microorganisms present in breast milk samples. Understanding the breast milk microbiome could have significant implications for maternal and infant health, particularly in areas such as immunity, nutrition, or microbiota-related therapies. These findings provide a foundation for future research exploring specific interactions between microbiota and health outcomes and their potential applications in different populations.

## Supplemental Materials

Supplementary data for this paper are available on-line only at http://jmb.or.kr.



## Figures and Tables

**Table 1:** Comparison of CT values for wall disruption in pretreated milk samples from healthy lactating women.

Option	1	2	3	4	5	6	7	8
M-mean	29.5	29.2	29.2	29	28.7	28.8	29.6	28.7
M-SD	5.5	5.7	5.8	5.9	5.8	6.1	6.3	6.1
V-mean	30.2	29.9	29.7	29.7	29.2	28.9	28.9	29
V-SD	5.7	5.8	6.2	5.9	6.4	6.4	6.5	6.7

M: M tube; V: V tube.

**Table 2:** Microbial reads of reference standard into healthy breast milk at different dilution gradients.

	NC	Matrix (M)	Spiked Sample(M)		
			L	M	H
*Pseudomonas aeruginosa*	-	40	306	8020	558661
*Escherichia coli*	-	65	59	6314	604165
*Salmonella enterica*	-	3195	2079	7257	509398
*Lactobacillus fermentum*	-	-	8	658	57650
*Enterococcus faecalis*	-	-	27	1580	42893
*Staphylococcus aureus*	-	19938	10275	6705	7793
*Listeria monocytogenes*	-	-	-	309	25439
*Bacillus subtilis*	-	-	-	-	150472
*Saccharomvces cerevisiac*	-	-	-	-	-
*Cryptococcus neoformans*	-	-	-	-	414

NC: Negative Control; L: low concentration; M: medium concentration; H: high concentration

**Table 3:** Alpha diversity indices for microbial communities in breast milk samples from 11 healthy mothers.

Participants	Chao1 Index	Species Number	Shannon Index
case 1L	86.4	67	2.55
case 1R	59	52	2.29
case 2L	159.5	140	3.33
case 2R	140.6	120	3.84
case 3L	183.2	160	3.88
case 3R	81	76	3.36
case 4L	164.2	128	3.35
case 4R	216.6	150	3.78
case 5L	93.3	80	2.87
case 5R	86.7	64	2.75
case 6L	85.7	76	1.16
case 6R	72.4	61	2.24
case 7L	84.6	80	0.41
case 7R	138.8	81	0.26
case 8L	80.2	71	2.63
case 8R	63	50	2.60
case 9L	82.1	65	0.23
case 9R	135.9	99	0.83
case 10L	168.3	111	2.27
case 10R	102.3	85	0.44
case 11L	69.1	59	1.25
case 11R	114.5	82	1.64

L: left; R: right.

**Table 4:** Comparative analysis of metagenomic sequencing results from healthy breast milk samples.