# Supplementary material for: Microbiome Profiling of Pretreated Human Breast Milk Using Shotgun Metagenomic Sequencing
Source: J Microbiol Biotechnol. 2025 Oct 28;35:e2506012. doi: 10.4014/jmb.2506.06012 (PMC12603376; doi:10.4014/jmb.2506.06012)
Supplement: Supplementary file 1 [file jmb-35-e2506012-supple.pdf]

J. Microbiol. Biotechnol. 2025. 35: e2506012

https://doi.org/10.4014/jmb.2506.06012

Table S1. Demographic and clinical characteristics of the study participants.

Subject	Age（y）	Feeding Mode	Delivery Mode
case1	39	Exclusive	Vaginal
case2	33	Exclusive	Cesarean
case3	30	Mixed	Vaginal
case4	33	Exclusive	Vaginal
case5	29	Exclusive	Vaginal
case6	32	Exclusive	Cesarean
case7	33	Mixed	Cesarean
case8	29	Mixed	Vaginal
case9	34	Mixed	Vaginal
case10	29	Mixed	Vaginal
case11	25	Exclusive	Vaginal

Exclusive: exclusive breastfeeding; Mixed: mixed feeding; Vaginal: spontaneous vaginal delivery; Cesarean: cesarean section.

Table S2. Gradients of the wall-breaking parameters in pretreatment sample.

**Parameter**	**Gradient**							
	**1**	**2**	**3**	**4**	**5**	**6**	**7**	**8**
**S（m/s）**	5	5	5	5	5.5	5.5	5.5	5.5
**T（s）**	40	50	40	50	40	50	40	50
**D（min）**	3	3	3	3	3	3	3	3
**C**	3	3	4	4	3	3	4	4

S： oscillation speed; T： running time; D： interval time; C： cycle number

Table S3. CT values of pretreatment samples.

	**Bacteria**	**M-1**	**M-2**	**V-1**	**V-2**		**Bacteria**	**M-1**	**M-2**	**V-1**	**V-2**
**1**	eal	22.42	22.42	22.89	22.93	5	eal	21.05	21.43	21.27	20.72
	sgc	32.91	32.88	33.12	33.47		sgc	31.33	32.06	32.61	32.14
	kpn	33.12	33.18	34.31	34.38		kpn	32.86	33.33	34.43	34.11
**2**	eal	21.53	22.16	22.69	22.23	6	eal	20.84	21.20	20.86	20.48
	sgc	31.75	32.40	33.18	33.53		sgc	31.30	31.64	31.89	31.76
	kpn	33.54	33.64	33.76	34.17		kpn	33.27	34.25	34.11	34.15
**3**	eal	21.50	22.02	21.83	21.64	7	eal	21.72	21.32	21.14	19.87
	sgc	32.33	32.12	32.99	32.78		sgc	32.57	32.36	32.98	31.95
	kpn	33.38	33.89	34.15	34.58		kpn	34.72	34.59	34.07	33.20
**4**	eal	21.50	21.56	22.31	22.09	8	eal	21.06	20.73	20.92	20.05
	sgc	32.23	31.85	33.15	32.37		sgc	32.20	31.22	32.79	31.76
	kpn	34.04	32.99	34.13	34.35		kpn	33.47	33.48	34.59	34.05

eal： Candida albicans； sgc： Streptococcus agalactiae； kpn： Klebsiella pneumoniae； M： M tube； V： V tube

Table S4. Summary of sequencing data for 11 samples.

Participants	Raw data (Mb)	Raw reads (M)	Clean data (Mb)	Clean reads (M)	Human sequence (%)	Non-human reads (M)	Q20 (%)	Q30 (%)
case 1L	6,261.08	20.73	4,563.99	19.63	92.19	1.53	95.95	91.16
case 1R	4,140.86	13.71	3,014.97	13.13	92.86	0.94	95.1	90.34
case 2L	4,407.71	14.6	3,188.18	14.02	92.74	1.02	95.88	91.07
case 2R	3,830.95	12.69	2,820.88	12.24	93	0.86	95.83	91.03
case 3L	5,159.98	17.09	3,809.69	16.19	94.04	0.96	95.65	90.99
case 3R	5,478.17	18.14	3,820.06	16.9	93.67	1.07	95.36	90.37
case 4L	3,623.74	12	2,966.36	11.76	93.17	0.8	96.04	91.33
case 4R	3,717.34	12.31	2,909.81	11.85	92.48	0.89	96.35	91.79
case 5L	3,535.04	11.71	2,732.03	11.3	93.4	0.75	95.88	91.17
case 5R	3,516.54	11.64	2,761.44	11.37	93.73	0.71	95.45	90.62
case 6L	8,689.24	28.77	6,710.94	28.1	93.96	1.7	96.62	92.34
case 6R	8,656.4	28.66	6,268.79	27.43	93.82	1.69	96.02	91.59
case 7L	8,245.88	27.3	6,191.34	26.45	83.2	4.44	96.12	91.73
case 7R	7,130.66	23.61	5,322.86	22.84	83.52	3.76	95.99	91.38
case 8L	5,187.07	17.18	3,583.85	15.69	94.2	0.91	94.94	90.27
case 8R	5,793.87	19.19	4,271.79	18.66	94.84	0.96	96.17	91.57
case 9L	5,520.47	18.28	4,104.96	17.6	93.62	1.12	96.49	92.32
case 9R	4,184.34	13.86	3,198.82	13.5	86.87	1.77	96.05	91.67
case 10L	6,362.17	21.07	4,751.8	20.47	89.33	2.18	96.4	91.94
case 10R	8,858.41	29.33	6,631.19	28.5	94.84	1.47	96.55	92.07
case 11L	5,102.73	16.9	3,657.88	16.16	95.43	0.74	96.44	92.07
case 11R	5,078.06	16.81	3,571.94	15.7	94.24	0.9	95.68	91.21

L: left; R: right.

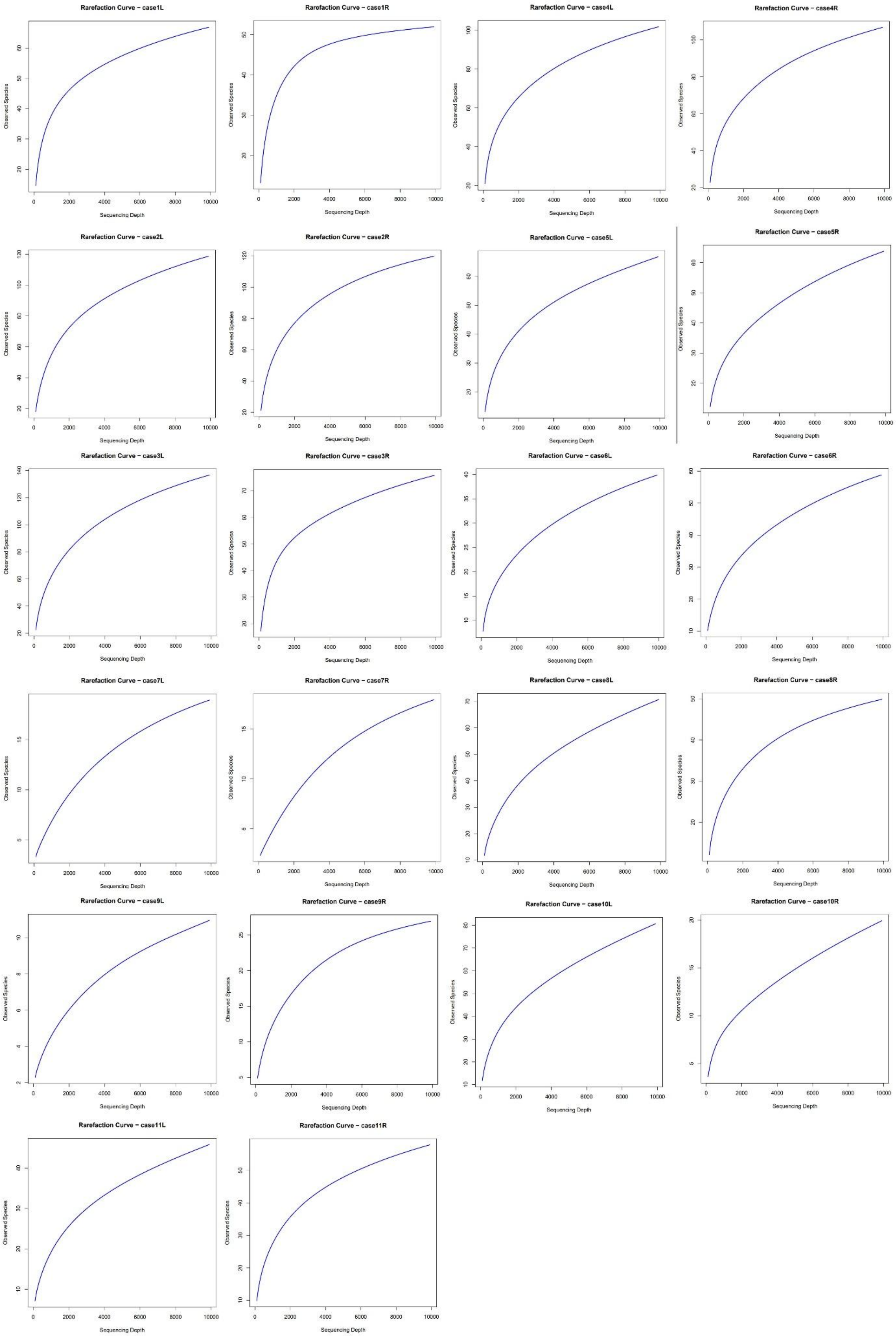
Rarefaction Curve – case1L
Rarefaction Curve – case1R
Rarefaction Curve – case4L
Rarefaction Curve – case4R
Rarefaction Curve – case2L
Rarefaction Curve – case2R
Rarefaction Curve – case5L
Rarefaction Curve – case5R
Rarefaction Curve – case3L
Rarefaction Curve – case3R
Rarefaction Curve – case6L
Rarefaction Curve – case6R
Rarefaction Curve – case7L
Rarefaction Curve – case7R
Rarefaction Curve – case8L
Rarefaction Curve – case8R
Rarefaction Curve – case9L
Rarefaction Curve – case9R
Rarefaction Curve – case10L
Rarefaction Curve – case10R
Rarefaction Curve – case11L
Rarefaction Curve – case11R
Observed Species
Sequencing Depth

Fig. S1. Rarefaction curves for all breast milk samples.

Curves were generated at the species level using subsampled sequencing reads. Most samples reached a plateau, indicating saturation of observed species.
